# Contribution of Baicalin on the Plasma Protein Binding Displacement and CYP3A Activity Inhibition to the Pharmacokinetic Changes of Nifedipine in Rats *In Vivo* and *In Vitro*

**DOI:** 10.1371/journal.pone.0087234

**Published:** 2014-01-30

**Authors:** Zhen-Yu Cheng, Xin Tian, Jie Gao, Hong-Meng Li, Lin-Jing Jia, Hai-Ling Qiao

**Affiliations:** Department of Clinical Pharmacology, School of Medicine, Zhengzhou University, Zhengzhou, China; Kyushu University, Japan

## Abstract

Baicalin purified from the root of *Radix scutellariae* is widely used in clinical practices. This study aimed to evaluate the effect of baicalin on the pharmacokinetics of nifedipine, a CYP3A probe substrate, in rats *in vivo* and *in vitro*. In a randomised, three-period crossover study, significant changes in the pharmacokinetics of nifedipine (2 mg/kg) were observed after treatment with a low (0.225 g/kg) or high (0.45 g/kg) dose of baicalin in rats. In the low- and high-dose groups of baicalin-treated rats, *C*_max_ of total nifedipine decreased by 40%±14% (*P*<0.01) and 65%±14% (*P*<0.01), AUC_0–∞_ decreased by 41%±8% (*P*<0.01) and 63%±7% (*P*<0.01), V_d_ increased by 85%±43% (*P*<0.01) and 224%±231% (*P*<0.01), and CL increased by 97%±78% (*P*<0.01) and 242%±135% (*P*<0.01), respectively. Plasma protein binding experiments *in vivo* showed that *C*_max_ of unbound nifedipine significantly increased by 25%±19% (*P*<0.01) and 44%±29% (*P*<0.01), respectively, and there was a good correlation between the unbound nifedipine (%) and baicalin concentrations (*P*<0.01). Furthermore, *in vitro* results revealed that baicalin was a competitive displacer of nifedipine from plasma proteins. *In vitro* incubation experiments demonstrated that baicalin could also competitively inhibit CYP3A activity in rat liver microsomes in a concentration-dependent manner. In conclusion, the pharmacokinetic changes of nifedipine may be modulated by the inhibitory effects of baicalin on plasma protein binding and CYP3A–mediated metabolism.

## Introduction

Plasma protein binding plays an important role in the whole-body disposition of drugs. Pharmacokinetic properties, such as distribution volume, hepatic metabolism, renal excretion and membrane transport, are highly related to the unbound fraction of drugs [1]. Some studies have shown that the displacement of drugs from plasma proteins can cause significant changes in their pharmacokinetics [2]–[4]. Among the CYP450 enzymes, CYP3A is the most abundant isoform, which metabolises more than 50% of drugs used in clinical practices, including midazolam, nifedipine and cyclosporin A [5]–[8]. Clinical and preclinical studies have reported that herbs such as *Sophora flavescens* and *St John’s Wort* could inhibit CYP3A activity and cause herb-drug interactions [9], [10].

Baicalin (BA, 5, 6-Dihydroxy-flavone-7-O-glucuronide) is the principal component purified from the root of *Radix scutellariae* and is regarded as the marker compound for quality control of over 100 examples of compound preparations in Chinese Pharmacopoeia. Extensive studies have revealed that baicalin exhibits strong anti-oxidant [11], anti-inflammatory [12], and hepato-protective [13], [14] activities. Baicalin is also a component in a wide range of vegetables, fruits, and beverages derived from plants [15], [16]. The widespread use of baicalin has led to the assessment of its safety and efficacy for human applications.

As natural vehicles for many types of endogenous and exogenous agents, plasma proteins are responsible for determining the pharmacokinetic properties of many drugs. Tang Y et al [1] showed that the plasma protein binding of baicalin was within the range of 86%–92% and the association constant (K_A_) was determined as 1.21×10^5^ L/mol. A high protein bound drug will typically have a K_A_ value ranging from 10^5^ to 10^7^ L/mol [17]. Liu H et al [18] reported that when administered with other Sudlow site I drugs (e.g., warfarin), baicalin could be converted into a relatively high-affinity binder of plasma albumin *in vivo*. Baicalin may also displace other drugs from the binding sites and enhance the potencies, which can be toxic.

Increasing attention has been paid to the effects of baicalin and other main bioactive constituents of *Radix scutellariae* on CYP450 enzymes. Recent studies have indicated that baicalin significantly induced CYP2B6-catalysed bupropion hydroxylation but had no effect on either CYP3A4 or MDR1 gene expression [19], [20]. Our preliminary studies revealed that baicalin inhibited the metabolism of dextromethorphan and midazolam, the recommended probe drugs for CYP2D and CYP3A, respectively, *in vivo* and *in vitro* in rats [21], [22]. However, we found that the effects and mechanisms of baicalin on CYP2E1 were different from those of CYP2D and CYP3A; baicalin inhibited CYP2E1 *in vitro*, but exerted no effect on the AUC and CL of chlorzoxazone in rats [23]. Importantly, the interactions observed with one CYP3A4 probe substrate may not be representative of those observed with other CYP3A4 substrates because CYP3A enzymes are known to accommodate multiple ligands in the active site. This may significantly affect the extrapolation of drug interactions from the *in vitro* to *in vivo* context or from one CYP3A4 substrate to another *in vitro* or *in vivo* [24], [25]. Thus, more than one probe drug was used to investigate drug interactions *in vivo* [26], [27].

Nifedipine is a typical dihydropyridine calcium channel blocker with predominant vasodilatory activity and is used widely in the treatment of hypertension and angina [28]. Previous *in vivo* and *in vitro* studies have indicated that nifedipine is a representative substrate of CYP3A [8], [29]–[31] and shows a different substrate behaviour compared with midazolam and testosterone [25]. Nifedipine binds highly to plasma proteins, and thus even small changes in protein binding are capable of producing marked changes in its pharmacokinetics [32]–[34]. On the basis of these data, our study was performed to determine the effect of different doses of baicalin on the pharmacokinetics of nifedipine, and the correlation between the free fraction of nifedipine and baicalin concentrations *in vivo*. Moreover, we also examined the effects of baicalin on the protein binding of nifedipine and CYP3A activity *in vitro* to identify the underlying mechanisms of these *in vivo* results.

## Materials and Methods

### Ethics Statement

This study was performed according to the Guide for the Care and Use of Laboratory Animals. All experimental procedures reported herein were reviewed and approved by the Zhengzhou University Animal Care and Use Committee.

### Drugs and Materials

Baicalin (>98.5% purity) was received as a gift from Henan Provincial Institute of Food and Drug Control. Nifedipine was purchased from the National Institute for the Control of Pharmaceutical and Biological Products (Beijing, China). NADPH was obtained from Roche Co. Ltd. (Switzerland). Diazepam injections were purchased from Tianjin Jin Yao Amino Acid Co., Ltd. (China). Ultrafiltration tubes (0.5 ml, 10KD) were purchased from Millipore (USA). All organic solvents of HPLC purity were obtained from Siyou Chemical Reagent Co. (Tianjin, China).

### Animals

Male Sprague Dawley rats (200–250 g) were obtained from the Laboratory Animal Center of Henan Province (Henan, China). Rats were housed in a temperature-controlled colony room under a 12 h light/dark cycle and had free access to food and water for 1 week prior to experiments. Rats were fasted overnight prior to the experiment, and given free access to water.

### Pharmacokinetic Studies of Nifedipine

The baicalin solution for the injections was prepared by dissolving 250 mg baicalin in 50 ml of Na_2_HPO_4_ (0.2 M) and adjusting to pH 7.4 with citric acid (0.1 M). The nifedipine solution for the injections were prepared by dissolving 20 mg of nifedipine in a mixture of polyethylene glycol 400 (5 ml) and saline (10 ml) according to Mohri K et al [35] with slight modifications.

Twelve rats were randomly divided into 3 groups (n = 4, each group), and the order of the baicalin doses was administered according to a Latin-Square design (saline, 0.225 and 0.45 g/kg) with a 3-day wash-out period between treatments. All pharmacokinetic data were obtained from 12 animals. In the pharmacokinetic studies, the rats were treated either with saline, low (0.225 g/kg) or high (0.45 g/kg) doses of baicalin. Nifedipine was administered immediately following the injection of baicalin or saline via the tail vein. In the pharmacokinetic studies, blood samples (300 µl) for pharmacokinetic analyses were collected pre-dose and at 0, 0.167, 0.5, 1, 2, 3 and 4 h post-nifedipine dose by orbital bleeding via heparinised capillary tubes. The plasma was obtained by centrifugation at 4,500 rpm for 10 min at 4°C and frozen at −80°C prior to analysis.

### HPLC Analysis of Nifedipine and Baicalin

The concentration of nifedipine in the blood samples was determined by a slight modification of a previously reported high-performance liquid chromatography (HPLC) method with gradient elution (63%–90% methanol in water) and UV detection at 235 nm [36]. Briefly, 10 µl of diazepam (0.09 mg/ml) as internal standard was added to a blood sample, which was then alkalinised by 15 µl of ammonia and subjected to liquid–liquid extraction using 2 ml ether. After vortex mixing for 2 min and centrifuging at 3,000 rpm for 10 min, 1.6 ml of the organic phase was transferred into another glass centrifuge tube and evaporated to dryness at 40°C under a gentle stream of nitrogen. The residue was reconstituted with 100 µl of mobile phase, and 40 µl was injected into the HPLC system (Agilent 1100 Series) for analysis. The quantitation limits of nifedipine in the rat plasma samples were 0.20 mg/L. All operations were performed under weak red light.

The concentration of baicalin in the blood samples was determined using a slightly modified high-performance liquid chromatography (HPLC) protocol with UV detection at 278 nm [37]. Briefly, 100 µl methanol was added to 25 µl of the blood sample. The mixture was vortex for 1 min after which it was centrifuged at 15,000 rpm for 10 min at 4°C, and 5 µl supernatant was then injected into the HPLC system (Agilent 1100 Series) for analysis. The quantitation limits of baicalin in the rat plasma were 5.86 mg/L.

### Plasma Protein Binding of Nifedipine *in vivo* and *in vitro*

For the *in vivo* study, the protein-binding of nifedipine in plasma at different sampling times after treatment with baicalin (0.225, 0.45 g/kg, iv) were evaluated using ultrafiltration. Plasma samples containing nifedipine and baicalin were prepared *in vitro*. The concentrations of nifedipine were from 13.0 to 52.0 mg/L, and the concentrations of baicalin were from 0 to 2000.0 mg/L. The resulting mixture was subsequently incubated at 37°C for 30 min, and 0.2 ml aliquots were placed into an ultrafiltration device (Millipore, USA). After centrifuging at 2,000 rpm for 20 min, concentrations of nifedipine were measured using HPLC.

### Effects of baicalin on CYP3A Activity *in vitro*

The preparation of rat liver microsome (RLM) suspensions were performed as previously reported [38]. The effect of baicalin on CYP3A activity was evaluated by measuring the metabolic velocity of nifedipine in RLMs. Briefly, the incubation mixtures (total volume 0.2 ml) contained microsomal protein (0.25 mg/ml), phosphate buffer (100 mM, pH 7.4), MgCl_2_ (3 mM), NADPH (1 mM), EDTA (0.1 mM), nifedipine (9.38–150 µM) and baicalin (12.5–200 µM). The reaction time was 30 min with a pre-incubation of 5 min without the addition of NADPH and was terminated by adding acetonitrile (20 µl). The mixture was vortex for 1 min after which it was centrifuged at 15,000 rpm for 10 min, and 20 µl supernatant was then injected into the HPLC system for analysis. The kinetic constants (K_m_ and V_max_) for the disappearance of nifedipine and inhibition constants (K_i_) were calculated using the nonlinear regression method. The quantitation limits of nifedipine in the RLM samples were 2.34 µmol/L.

### Data Analysis

The pharmacokinetic parameters were determined using a non-compartmental pharmacokinetic model with the DAS 2.0 package (version 2.0 pharmacokinetic software; Chinese Pharmacological Assn., Beijing, China). Michaelis-Menten Enzyme kinetics data were fitted using non-linear regression analysis with GraphPad Prism 5 (GraphPad Software Inc., CA, USA). The mechanism of inhibition was determined by visual inspection of the data using a Lineweaver-Burke (1/[S] vs. 1/v) plot. The K_i_ was obtained using the secondary plot of the Lineweaver-Burk plot. The peak plasma concentration of the total and unbound nifedipine was obtained from actual data (t = 0 h). The data of *C*_max_, AUC and CL were analysed using the paired *t*-test. Correlations were measured using Pearson's correlation coefficient and Spearman's Rho. The results are expressed as the mean ± SD. A value of *P*<0.05 was considered to be statistically significant. All statistical analyses were performed with *SPSS* 17.0 for Windows.

## Results

### Effects of baicalin on the Pharmacokinetics of nifedipine in Rats

#### The pharmacokinetics of nifedipine in rats

**Figure 1:**
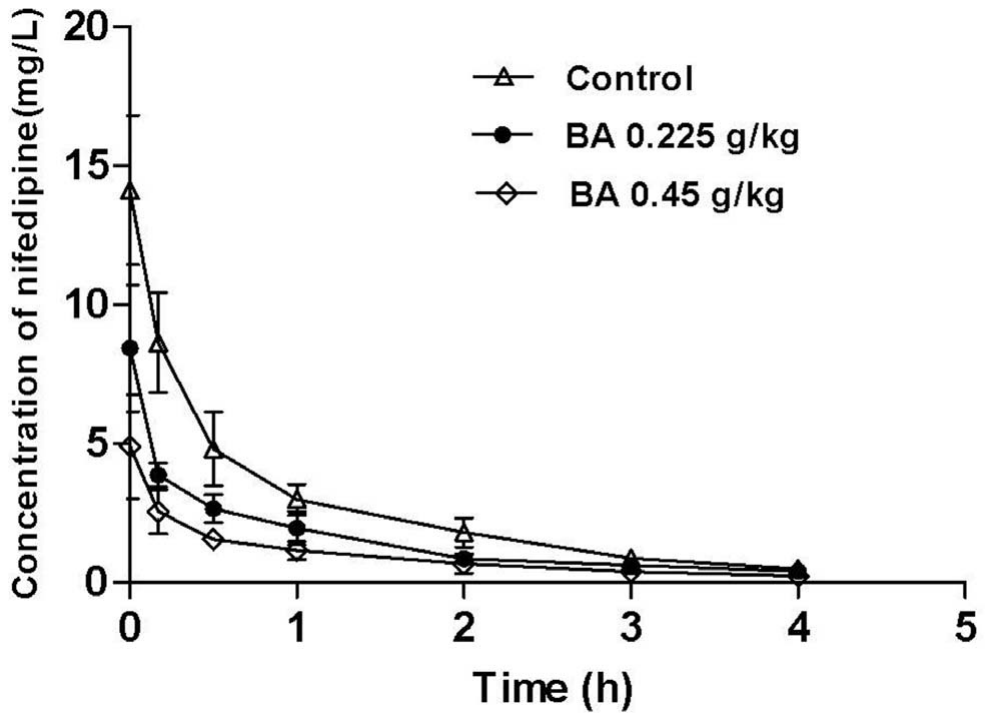
The plasma concentration-time profiles of total nifedipine (2 mg/kg, i.v.) after treatment with baicalin (0.225, 0.45 g/kg) in rats (mean ± SD, n = 12).

The mean plasma concentration–time profiles of total nifedipine after the intravenous administration of nifedipine (2 mg/kg, i.v.) with saline (control) or baicalin (0.225, 0.45 g/kg) are shown in Figure 1. The key pharmacokinetic parameters of nifedipine are summarised in Table 1. These results showed that after treatment with baicalin (0.225, 0.45 g/kg), the maximum concentrations (*C*_max_) of total nifedipine decreased by 40%±14% and 65%±14% (*P*<0.01), area under plasma concentration–time curve (AUC_0–∞_) decreased by 41%±8% and 63%±7% (*P*<0.01), apparent volume of distribution (V_d_) increased by 85% ±43% and 224%±231% (*P*<0.01), and clearance (CL) increased by 97%±78% and 242%±135% (*P*<0.01). These observations strongly indicated that baicalin significantly altered the pharmacokinetics of nifedipine in rats.

**Table 1:** Pharmacokinetics parameters of nifedipine (2 mg/kg, i.v.) after treatment with baicalin (0.225, 0.45 g/kg, i.v.) in rats (n = 12).

	Control	Baicalin (0.225 g/kg)		Baicalin (0.45 g/kg)	
	Value	Value	Ratio	Value	Ratio
*C*_max_ (mg/L)	14.12±2.70	8.42±2.28**	0.60±0.14	4.97±1.75** ^△△^	0.35±0.14
T_1/2_(h)	0.40±0.17	0.26±0.07		0.46±0.22	
V(L/kg)	0.14±0.03	0.26±0.06**	1.85±0.43	0.46±0.07** ^△^	3.24±2.31
CL(L/h/kg)	0.27±0.07	0.54±0.12**	1.97±0.78	0.94±0.34** ^△^	3.42±1.35
AUC_0–t_(mg·h/L)	10.43±1.35	5.87±1.10**	0.57±0.09	3.66±0.63** ^△△^	0.35±0.07
AUC_0–∞_(mg·h/L)	11.14±1.17	6.58±1.29**	0.59±0.08	4.06±0.67**	0.37±0.07

vs. control,

* *P*<0.05,

** *P*<0.01.

vs. baicalin (0.225 g/kg, i.v.),

△ *P*<0.05,

△△ *P*<0.01.

#### Individual variability of nifedipine pharmacokinetic changes

**Figure 2:**
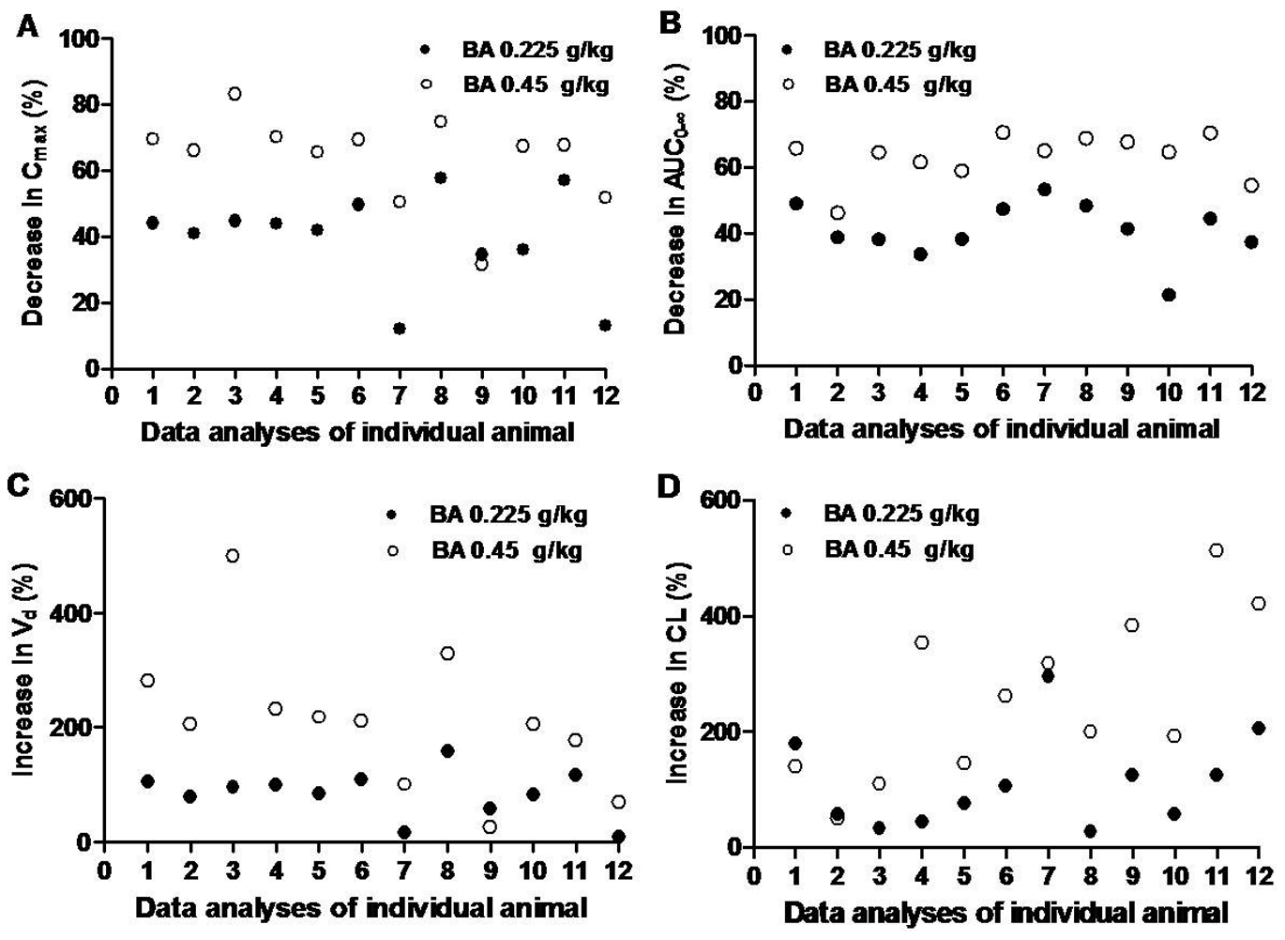
The inter-individual differences in the changes induced by baicalin (0.225, 0.45 g/kg) in the pharmacokinetic parameters of nifedipine (2 mg/kg) (mean ± SD, n = 12).

As shown in Figure 2A, a significant decrease in *C*_max_ of nifedipine occurred after treatment with baicalin. However, the *C*_max_ of rat 7 decreased by 12.11%, while that of rat 8 decreased by 57.91% when the rats received baicalin at a dose of 0.225 g/kg. Moreover, there was a nearly 3-fold difference in *C*_max_ at a dose of 0.45 g/kg. In addition, corresponding AUC_0–∞_, V_d_ and CL variations were also observed (Figure 2B, C and D). Taken together, the data showed that there were large inter-individual differences in the nifedipine-baicalin interactions.

#### Relationship between the concentration changes of nifedipine and the concentrations of baicalin

**Figure 3:**
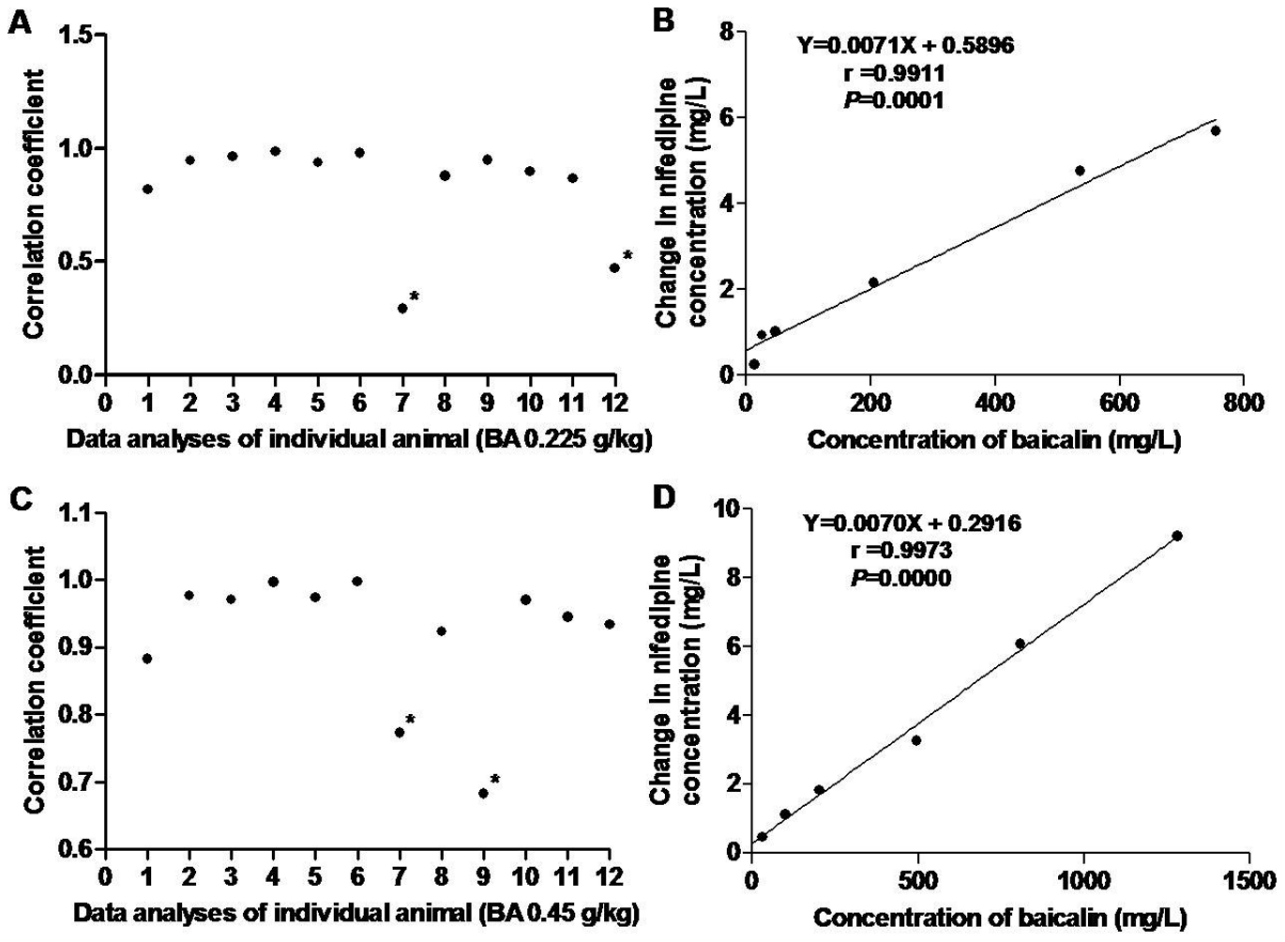
Relationship between changes in nifedipine concentrations and corresponding baicalin concentrations in rats. (A), (C) Correlation coefficient of changes in nifedipine concentration in different sampling times and corresponding baicalin concentrations in rats. (B) (D) Plots of the mean changes in nifedipine concentrations in rats treated with baicalin (0.225, 0.45 g/kg) versus mean baicalin concentrations of corresponding doses (n = 12). *P>0.05 in correlation analysis.

The pharmacokinetic parameters of baicalin at doses of 0.225 and 0.45 g/kg were examined (data not shown). We discovered that there were significant correlations except in two rats treated with baicalin at 0.225 g/kg and another two rats treated with baicalin at 0.45 g/kg (Figure 3A and C). The correlations between the mean changes in nifedipine concentrations and mean baicalin concentrations in rats are shown in Figure 3B and 3D. The coefficients (r) were 0.9911 and 0.9973, respectively.

### Plasma Protein Binding of nifedipine *in vivo* and *in vitro*

**Figure 4:**
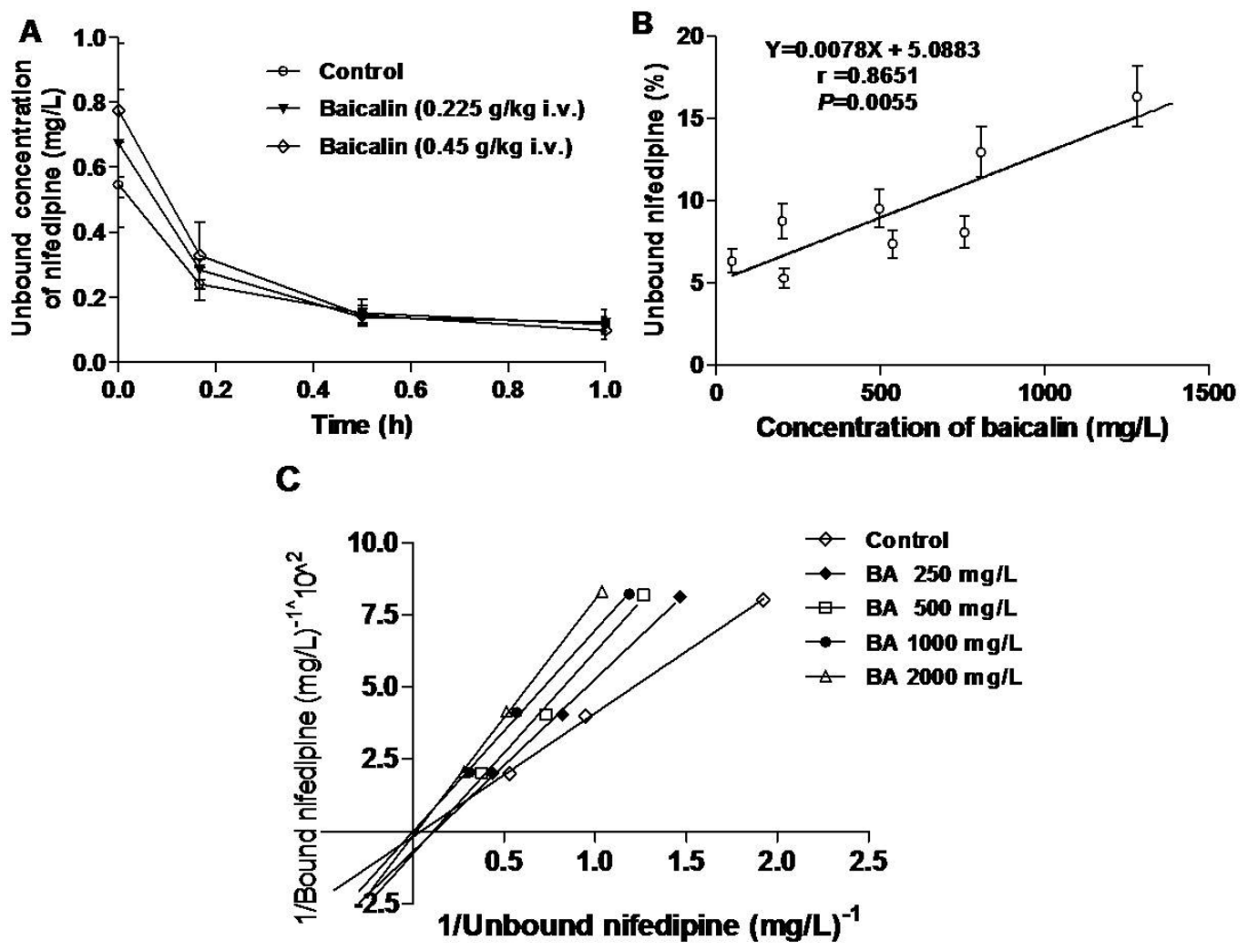
Interaction of nifedipine and baicalin for plasma proteins *in vivo* and *in vitro*. (A) The plasma concentration-time profiles of unbound nifedipine (2 mg/kg, i.v.) after treatment with baicalin (0.225, 0.45 g/kg) in rats. (B) Correlation between unbound nifedipine (%) and corresponding baicalin concentrations in rats. (C) Double reciprocal plot of the molar concentration of unbound versus bound nifedipine in plasma. The plasma nifedipine concentration ranged from 13 to 52 mg/L.

To examine the interaction between baicalin and nifedipine *in vivo*, we examined the unbound fraction of nifedipine at the sampling time 0, 0.167, 0.5 and 1 h. As shown in Figure 4A, the results showed that the *C*_max_ of unbound nifedipine was significantly increased by 25%±19% and 44%±29% (*P*<0.01), respectively. An *in vivo* protein binding study also revealed detailed changes of unbound nifedipine (%) in the pharmacokinetic samples for nifedipine when treated with saline or baicalin. The unbound nifedipine (%) values after treatment with baicalin (0.225, 0.45 g/kg) were 8.09% and 16.06% at 0 h, and the mean unbound nifedipine (%) after treatment with nifedipine and saline was 3.92%.

A positive rank order correlation between unbound nifedipine (%) and corresponding baicalin concentrations in rats (r = 0.8651) is shown in Figure 4B. These results demonstrated a good prediction of unbound nifedipine (%) from the baicalin concentration value.

The present assay has been successfully applied to quantify the concentration of nifedipine in rat plasma in drug–protein binding studies in the presence or absence of baicalin using ultrafiltration *in vivo* and *in vitro*. The double reciprocal plot for unbound nifedipine concentrations (%) to plasma protein in the absence and presence of baicalin at concentrations of 250, 500, 1000, 2000 mg/L is shown in Figure 4C. These results clearly illustrated that the interactions between nifedipine and baicalin for rat plasma proteins were competitive, and the unbound nifedipine (%) significantly increased from 4.00% to 8.28% with increasing concentrations of baicalin.

### Effects of baicalin on CYP3A Activity

**Figure 5:**
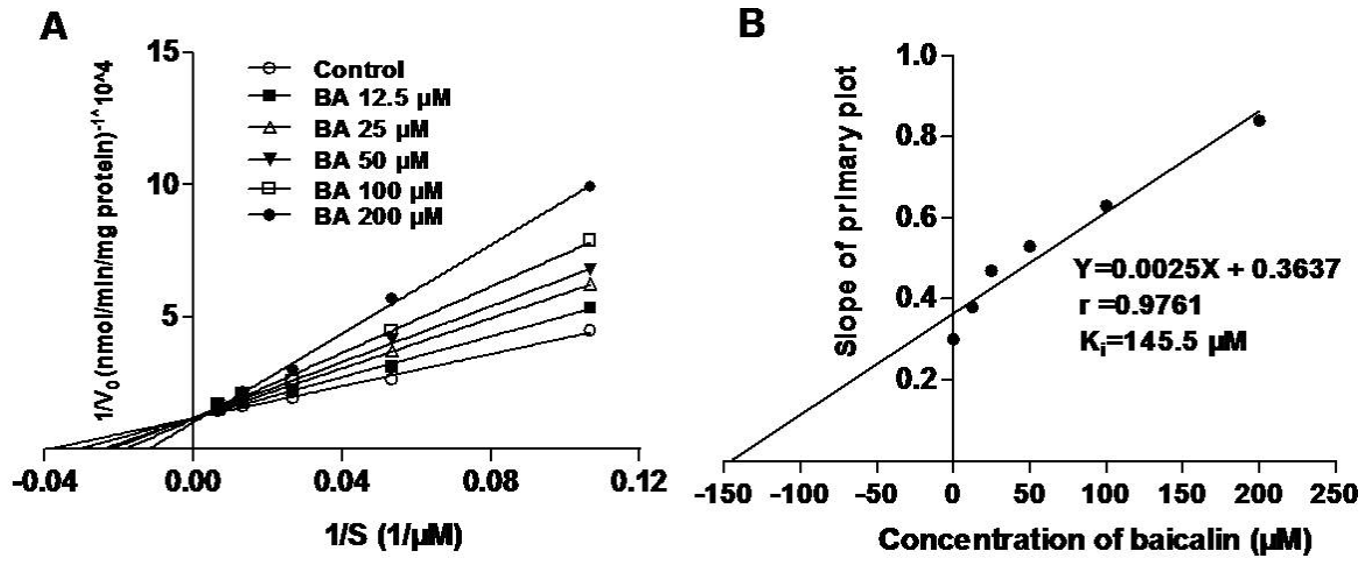
Effects of baicalin on CYP3A activity *in vitro*. (A) Primary Lineweaver-Burke plots were used to plot the effect of baicalin on nifedipine metabolism in RLMs. (B) Secondary plots of rat CYP3A activity using the slopes of primary Lineweaver-Burke plots versus the concentration of baicalin.

To investigate the kinetics of the inhibitory effects of baicalin on hepatic CYP3A activity, nifedipine disappearance in RLMs was examined in the presence and absence of baicalin *in vitro*. In the absence of baicalin, the K_m_, V_max_, and CL_int_ of nifedipine in RLMs were 24.25 µM, 8.04 nmol/min/mg protein and 0.33 ml/min/mg protein, respectively. An inhibition study was performed at various concentrations of baicalin (Figure 5). The intersection point of these lines corresponds to each baicalin concentration and was close to the y-axis (Figure 5A). It has been suggested that the inhibition of CYP3A by baicalin was best fit in a competitive manner. The K_i_ value was calculated from second plot of the slopes derived from the Lineweaver-Burk plots vs. the concentrations of baicalin and was 145.5 µM (64.9 mg/L) (Figure 5B).

## Discussion

In recent years, there has been growing interest in herb-drug interactions, as they can potentially cause toxicity and/or attenuate drug efficacy in clinical treatment. Baicalin, as a marker compound for many herb medicines, is a high protein bound drug and inhibitor of CYP450s [18], [23]. It has been speculated that the herb-drug interaction may occur when baicalin is co-administered with other drugs.

Nifedipine, a substrate of CYP3A with high protein binding [39], [40], is widely used in the treatment of hypertension and angina. Hypertensive patients require long-term health care, and nifedipine is commonly co-administered with other drugs, such as baicalin. The interaction between baicalin and nifedipine was performed in this study to investigate the effects of baicalin on pharmacokinetics *in vivo* and metabolism in an *in vitro* incubation system of nifedipine.

After the intravenous co-administration of nifedipine and baicalin (0.225, 0.45 g/kg), the mean values of AUC of nifedipine were significantly lower compared to treatment without baicalin by approximately 41% and 63%, respectively. The mean values of CL were significantly enhanced by approximately 97% and 242%, respectively. These data showed that co-administration of baicalin markedly contributed to the changes in the pharmacokinetics of nifedipine.

The V_d_ and CL of drugs with high plasma protein binding capacity, such as nifedipine, were affected by other drugs [4], [41]. Because both nifedipine and baicalin are highly bound to plasma proteins, it is conceivable that baicalin could displace nifedipine from binding sites, resulting in changes in the pharmacokinetics of nifedipine. The displaced nifedipine from the plasma protein sites would then redistribute to the extravascular space with an increase in V_d_ and decrease in *C*_max_ of total nifedipine. The unbound fraction of nifedipine was increased despite the significant decrease in total nifedipine after baicalin treatment (Figure 4A). Interestingly, the unbound nifedipine (%) in rats treated with baicalin (0.225, 0.45 g/kg) was increased nearly four times more than that of control, varying from 3.92% to 16.06%. This type of displacement has been demonstrated in our previous study where baicalin, as a potential displacer for plasma protein binding sites, exhibited significantly increased unbound chlorzoxazone concentrations *in vivo* in rats [23]. The intrinsic clearance (CL_int_) was calculated from CL_total_/f_u_, where f_u_ is the unbound-fraction of nifedipine in plasma, and CL_int_ is directly related to the activities of the enzymes, which remove drugs from the body [42]. Thus, the increase in CL_total_ is due to the increase in the unbound fraction of nifedipine. Furthermore, an *in vivo* protein binding study also showed that there was a good correlation between unbound nifedipine (%) and corresponding baicalin concentrations in rats (r = 0.8651) after treatment with baicalin. This finding demonstrated a good prediction of the baicalin effect on unbound nifedipine (%) from the baicalin plasma concentration value. Moreover, there was no change in nifedipine T_1/2_ associated with baicalin administration, potentially because the T_1/2_ in a two compartment system is a ‘hybrid’ parameter that reflects drug clearance and the apparent volume of distribution [43]. In this study, baicalin not only inhibited CYP3A, but it also significantly increased the unbound concentration and decreased the AUC of nifedipine in rats. These results differed from the inhibitory effects of baicalin on other CYPs in our previous studies [21]–[23], suggesting that baicalin confers multiple effects on the activities of different CYPs and the pharmacokinetics of probe drugs via various mechanisms.

To determine if the interactions between baicalin and nifedipine in these cases resulted from a competition for plasma proteins, an *in vitro* protein binding study was performed. We discovered that baicalin competitively displaces nifedipine from plasma protein binding sites as demonstrated by the change in slope of the double reciprocal plot (Figure 4C) obtained from the *in vitro* plasma protein binding experiment [44]. The *C*_max_ values of baicalin in rats treated with baicalin (0.225, 0.45 g/kg) were 754.8 mg/L and 1,280.4 mg/L, respectively, and when the concentration of baicalin was increased above 250 mg/L, the unbound nifedipine significantly increased in the *in vitro* study (Figure 4C).

The current study assessed the inter-individual variations in drug distribution and metabolism using a self-controlled rat model (Figure 3). We observed the increase in V_d_, which ranged from 25% to 765% and in CL, which ranged from 50% to 513%. These results indicated that there was a large difference in the effects of baicalin on the metabolism of nifedipine in rats. Furthermore, it is well known that inter-individual differences in pharmacokinetics are much greater in humans compared to experimental animals [45]. Nifedipine is a drug that shows extremely broad inter-individual metabolic differences in human [46], [47]. Thus, additional studies are warranted to examine the interaction between baicalin and nifedipine in clinical practices.

Baicalin has been shown to inhibit the CYP3A-mediated metabolism of midazolam and cyclosporine *in vivo* [22], [48]. However, in the present study, baicalin did not demonstrate a direct inhibition on the pharmacokinetics of nifedipine. To explore whether baicalin can inhibit the metabolism of nifedipine, an *in vitro* incubation study in RLMs was performed in a subsequent study. These findings showed that the K_m_ and V_max_ values were consistent with previous observations [49], and baicalin was a relatively weak inhibitor of CYP3A in RLMs, with a competitive inhibitory effect. However, the pharmacokinetics of baicalin studies showed that the *C*_max_ values of baicalin in rats treated with baicalin at 0.225 g/kg and 0.45 g/kg were 754.8 mg/L and 1280.4 mg/L, respectively. Nifedipine is a low extraction drug with ER ranges from 0.22 to 0.32 [50]. For low hepatic extraction ratio drugs, the magnitude of an *in vivo* drug–drug interaction obtained from the inhibition of metabolic clearance can be predicted using the ratio of the inhibitor concentration ([I]) to inhibition constant (K_i_) [51]. In this study, K_i_ was dramatically lower than [I]; thus, the effect of baicalin on nifedipine was noticeable. It is also well known that an increase in the unbound fraction of a drug by a displacer will increase its clearance based on the total plasma drug concentration, which in turn, may mask a concomitant effect of the displacer as an enzyme inhibitor in decreasing drug clearance [52], [53]. It has been speculated that the higher unbound concentrations might be sustained for the duration of baicalin therapy and cause lethargy, bradycardia, marked hypotension and a loss of consciousness in humans [54]. In summary, the significant change in the pharmacokinetics of nifedipine in baicalin-treated rats is due to the combined inhibitory activity of CYP3A and the displacement of nifedipine from plasma protein binding sites by baicalin.

In the present study, an increase in the unbound concentration and decrease in AUC of nifedipine in plasma were observed after co-administration of baicalin in rats. The higher unbound concentrations might be sustained for the duration of baicalin therapy because baicalin also decreased the intrinsic clearance of nifedipine *in vitro*. If the results could be extrapolated to humans, then modification of the regimens of nifedipine and baicalin might be required and appropriate strategies should be adopted to minimise the adverse drug reactions in clinical practices. However, the baicalin-nifedipine interaction requires further investigations in human due to species differences between rat and human.
